# Bisphenol F and Steatotic Liver Disease: Resolving the PXR Paradox Through Stress Pathway Mechanisms

**DOI:** 10.3390/biomedicines14010030

**Published:** 2025-12-22

**Authors:** Enwar Abdalkarim AbdalHussin, Zariyantey Abd Hamid, Muhd Hanis Md Idris, Maizatul Hasyima Omar, Izatus Shima Taib

**Affiliations:** 1 Centre for Diagnostics, Therapeutics and Investigative Studies, Faculty of Health Sciences, Universiti Kebangsaan Malaysia, Jalan Raja Muda Abdul Aziz, Kuala Lumpur 50300, Malaysia; p108509@siswa.ukm.edu.my; 2 Pathology Department, College of Veterinary Medicine, Dhi Qar University, Dhi Qar City 64001, Iraq; 3 Integrative Pharmacogenomics Institute (iPROMISE), Level 7, FF3 Building, UiTM Puncak Alam Campus, Puncak Alam 42300, Malaysia; muhdhanis@uitm.edu.my; 4 Phytochemistry Unit, Herbal Medicine Research Centre, National Institutes of Health (NIH), Persiaran Setia Murni, Setia Alam, Shah Alam 40170, Malaysia; maizatul@imr.gov.my

**Keywords:** hepatotoxicity, lipid metabolism, pregnane X receptor, stetaotic

## Abstract

Steatotic liver disease (SLD) represents a major global health burden, with environmental toxicants emerging as critical contributors alongside metabolic dysfunction. Bisphenol F (BPF), an increasingly prevalent replacement for bisphenol A, is widely detected in human biological samples and environment, yet its hepatotoxic mechanisms remain incompletely characterized. This review synthesizes current evidence on BPF-induced SLD, with a particular focus on resolving the “pregnane X receptor (PXR) paradox”, the mismatch between BPF’s weak direct activation of PXR and the PXR-like metabolic effects observed in vivo. Comprehensive analysis of mechanistic pathways reveals that BPF-induced SLD develops predominantly through PXR-independent mechanisms involving oxidative stress, endoplasmic reticulum dysfunction, Drp1-mediated mitochondrial fission, NLRP3/NF-κB-driven inflammation, dysregulated post-translational modifications, and epigenetic remodelling. These converging pathways collectively disrupt hepatic lipid metabolism, promote triglyceride accumulation, and establish a self-perpetuating cycle of metabolic dysfunction. Notably, weak indirect PXR modulation via oxidative stress represents a secondary, non-causal mechanism unsupported by functional validation. This framework distinguishes toxicant-induced steatosis from metabolic dysfunction-associated steatotic liver disease while highlighting critical evidence gaps—particularly the absence of causal PXR validation studies and human epidemiological data. Therapeutic opportunities exist at validated convergence points including mitochondrial dynamics (Drp1), inflammatory signalling (NLRP3/NF-κB), and energy metabolism (AMPK-mTOR), though combination strategies targeting multiple pathways will likely be required for durable disease reversal. These findings necessitate the expansion of regulatory screening paradigms to incorporate cellular stress pathway biomarkers alongside traditional nuclear receptor endpoints, ensuring comprehensive hepatotoxic risk assessment of emerging BPA substitutes.

## 1. Introduction

Liver diseases have become a major global health issue, resulting in nearly 1.26 million deaths annually from cirrhosis and other chronic liver conditions [1]. One type of liver disease is the metabolic dysfunction-associated steatosis liver disease (MASLD), which has rapidly emerged as the predominant chronic liver disease worldwide [2]. MASLD affects a substantial proportion of the global population and is increasing in parallel with the rising prevalence of obesity and diabetes [1]. Recent studies indicate that the progression of liver disease is not solely attributable to conventional risk factors such as alcohol or viral hepatitis, but is strongly influenced by environmental toxicants, including endocrine-disrupting chemicals such as bisphenol F (BPF) [3]. Wang et al. [4] found that serum BPF levels were higher in patients with liver disease compared to controls, suggesting a potential association between BPF levels and steatotic liver disease (SLD). Furthermore, growing evidence suggests that environmental toxicants, including BPF, significantly contribute to the onset and progression of SLD [5].

Mechanistic investigations demonstrate that BPF promotes SLD via several interrelated mechanisms. Oxidative stress and mitochondrial dysfunction, driven by aberrant mitochondrial fission that is regulated by dynamin-related protein 1 (Drp1), play a pivotal role in BPF-induced lipid droplet accumulation in hepatocytes [6]. Inflammatory responses further intensify liver damage, while BPF-induced post-translational modifications (PTMs) impair hepatic cellular function [6,7]. Epigenetic alterations, such as DNA methylation, histone modifications, and RNA regulation, induce long-term changes in gene expression without affecting the DNA sequence, making them a critical interface for BPF’s deleterious effects [8]. BPF also interferes with lipid metabolism by disrupting essential regulatory mechanisms, leading to hepatic steatosis [7].

Xenobiotic compounds commonly bind to nuclear receptors, such as the pregnane X receptor (PXR), constitutive androstane receptor (CAR), and farnesoid X receptor (FXR), to regulate the expression of drug-metabolizing enzymes involved in xenobiotic metabolism. PXR is a nuclear receptor involved in xenobiotic sensing and metabolic regulation [9]. Toxicants activate PXR, influencing the expression of enzymes and transporters involved in detoxification, thereby reducing the harmful effects of toxicants and altering metabolic pathways associated with liver steatosis and inflammation [10]. Beyond its classical role in detoxification, PXR also regulates bile acid metabolism, inflammatory responses, as well as lipid and glucose homeostasis [11]. Intriguingly, while PXR activation has been implicated in steatotic liver disease and BPF has been linked epidemiologically and experimentally to SLD, current evidence indicates that BPF-induced hepatic steatosis does not primarily operate through a canonical PXR-mediated pathway. This apparent discrepancy between the shared involvement of BPF and PXR in SLD and the lack of direct PXR dependence in BPF’s actions represents a central mechanistic paradox that remains unresolved.

Although BPF has been regarded as a safer substitute for BPA compared to bisphenol S (BPS) and bisphenol AF (BPAF) [12], it shares a similar chemical structure and exhibits comparable estrogen-mimicking activity. Its presence in environmental and human samples raises concerns about potential toxic effects similar to other bisphenol analogues. Despite increasing evidence of BPF’s impact on hepatic function, its precise mechanisms in inducing SLD remain unclear. This review aims to address these gaps by highlighting the mechanistic pathways underlying BPF-induced SLD and by critically examining the “PXR paradox,” namely, how BPF contributes to SLD in the context of a nuclear receptor that is strongly linked to steatosis yet appears not to be the primary driver of BPF’s hepatic effects.

## 2. Steatotic Liver Disease and Metabolic Dysfunction-Associated Steatotic Liver Disease

SLD is the current overarching term that encompasses all liver conditions involving abnormal fat accumulation (steatosis) within hepatocytes [13]. This terminology replaces the former label fatty liver disease and integrates a range of aetiologies, including metabolic, alcohol-related, and mixed causes of hepatic steatosis [14]. According to the American Association for the Studies of Liver Diseases (AASLD) (2023), histologically, SLD is defined by the presence of lipid deposition in at least 5% of hepatocytes, regardless of the underlying cause [13]. Globally, SLD represents one of the most prevalent chronic liver disorders, affecting roughly 25–30% of the adult population [15]. The disease displays a continuity, from simple hepatic fat accumulation to inflammation, fibrosis, cirrhosis, and hepatocellular carcinoma (HCC), when left untreated. This progressive nature underscores SLD’s major public health importance as a cause of liver-related morbidity and mortality worldwide.

The adoption of the SLD nomenclature provides a more inclusive and mechanistically accurate framework, removing the stigmatizing and exclusionary connotation of “non-alcoholic” and better reflecting the complex etiopathogenesis of hepatic lipid accumulation. Among its subclasses, MASLD constitutes the most common and clinically significant category. MASLD is defined by hepatic steatosis in individuals presenting with one or more cardiometabolic risk factors, such as obesity, insulin resistance, dyslipidemia, type 2 diabetes mellitus, or hypertension, in the absence of substantial alcohol intake [13,16]. The redefinition emphasizes metabolic dysfunction as the central pathogenic driver rather than the absence of alcohol use, aligning disease classification with modern metabolic research.

The pathophysiological mechanisms underlying MASLD involve a multifactorial network of insulin resistance, altered lipid metabolism, oxidative and endoplasmic reticulum (ER) stress, mitochondrial dysfunction, and chronic low-grade inflammation [17]. These processes lead to hepatic lipid accumulation, inflammatory cytokine release, and activation of fibrogenic pathways. The advanced inflammatory form, known as metabolic dysfunction–associated steatohepatitis (MASH) is characterized by hepatocellular ballooning, lobular inflammation, and progressive fibrosis that may culminate in cirrhosis or HCC [15]. MASLD is now understood to be not merely as a hepatic disorder but as a multisystem metabolic disease that is intricately linked to obesity, cardiovascular morbidity, and type 2 diabetes. This paradigm shift emphasizes the liver’s role as a central organ in systemic metabolic dysregulation. Lifestyle interventions, particularly weight reduction, enhancement of insulin sensitivity, and cardiometabolic risk management, remain the cornerstone of therapy, as resmetirom is currently the only approved pharmacological agent for MASH/MASLD with moderate to advanced fibrosis [15].

Beyond metabolic and alcohol-related aetiologies, there is growing evidence that sustained exposure to environmental and industrial toxicants can independently provoke hepatic lipid accumulation and inflammation, even in individuals lacking classical metabolic risk factors [18]. Histologically, such toxicant-induced liver injury shares features with metabolic forms of steatotic disease, including lipid accumulation, hepatocellular ballooning, inflammatory cell infiltration, and progressive fibrosis. Populations with prolonged occupational or environmental exposure, particularly to vinyl chloride, solvents, or pollutants such as bisphenols, phthalates, polychlorinated biphenyls, dioxins, organochlorine insecticides, polycyclic aromatic hydrocarbons, and heavy metals like cadmium and arsenic, exhibit higher risks of liver fat deposition and dysfunction [19,20]. These chemicals act through diverse mechanisms, including endocrine disruption, oxidative stress, mitochondrial impairment, and dysregulation of xenobiotic receptor signalling (e.g., PXR, CAR, FXR, and PPAR pathways) [10] and lipid metabolism pathways. Because many of these pollutants persist in the environment and bioaccumulate in the human body through ingestion, inhalation, dermal contact, or occupational exposure, they represent a growing concern for liver health and a potential cofactor accelerating the progression of SLD [19].

## 3. Bisphenol F

**Figure 1:**
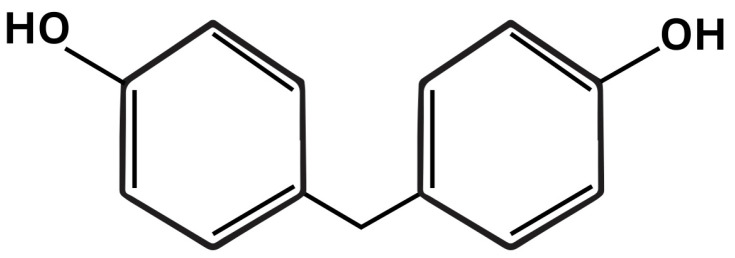
The chemical structure of BPF.

BPF has been employed as an alternative to BPA. BPF, also known as *4,4′-methylenediphenol*, is an organic compound that is a critical element of the bisphenol family of industrial chemicals [21]. BPF consists of two para-substituted phenol rings linked by a methylene (-CH_2_-) bridge, exhibiting physicochemical features identical to those of BPA (Figure 1) [22]. The molecular weight is approximately 200.23 g/mol, and the chemical formula is C_13_H_12_O_2_. BPF is a white to off-white crystalline solid with limited solubility in water under typical conditions. It is, nevertheless, very soluble in several organic solvents, such as acetone, chloroform, and ethanol [22,23].

BPF is frequently utilized in the production of epoxy resins and certain plastics, typically serving as a replacement for BPA in “BPA-free” items [24]. BPF has been utilized in several applications, such as structural adhesives, grouts, coatings, electrical varnishes, industrial floors, tank and conduit linings, and sealants for road and bridge decks [25]. The widespread use of BPF has resulted in its presence in supermarket products such as canned foods, meat, fish, shellfish, dairy items, vegetables, and fats and oils [26]. Moreover, BPF has also been detected in soil, surface water, and sewage [27]. Research on environmental degradation reveals that BPF experiences photodegradation in aquatic settings and biodegradation in the presence of hydroxyl radicals [28]. It has reduced volatility compared to BPA, hence diminishing its probability of atmospheric infiltration. However, its accumulation in sediments and persistence in aquatic habitats provide substantial threats to the food chain and marine animals [29]. BPF was detected in residential dust samples in the United States, where approximately 68% of BPF was identified in indoor dust samples obtained from households in New York [30].

Li et al. [3] stated that individuals may be exposed to BPF by the ingestion of contaminated food and beverages, inhalation of contaminated dust or air, or dermal contact with consumer products containing BPF. BPF has been detected in human biological specimens, such as blood serum and adipose tissue, demonstrating its capacity to persist in lipid-rich environments [25]. It has been shown that BPF is efficiently absorbed after oral administration in rats. However, research indicates that BPF is excreted more efficiently via urine compared to BPA. This may affect the bioaccumulation patterns of BPF in organisms [31]. Furthermore, residual doses were identified in many organs, such as the liver, gastrointestinal lumen, placenta, amniotic fluid, uterus, and fetuses of pregnant rats [32].

The molecular resemblance between BPF and BPA implies that both substances may have comparable toxicological mechanisms. Nevertheless, BPF’s unique metabolic characteristics heighten concerns regarding its prolonged effects on human health [33]. Increasing evidence indicates that BPF interacts with estrogen and other nuclear hormone receptors, leading to endocrine disruption that may affect metabolic, developmental, and reproductive processes. Gestational exposure to BPF modified the behaviour of mouse offspring [34]. The U.S. Environmental Protection Agency (EPA) designated BPF as a notable developmental hazard and a moderate reproductive hazard, based on the toxicity of analogous chemicals, and identified it as a considerable risk for toxicity from repeated exposures [35]. Recent findings indicate that BPF may interfere with the endocrine system by acting as both an estrogenic and antiandrogenic agent, leading to metabolic disruptions and reproductive damage [36]. In vitro studies have shown that BPF possesses both estrogenic and anti-androgenic characteristics similar to BPA [37,38]. BPF exposure has been linked to reduced testosterone levels in males and compromised sperm motility, highlighting its possible impact on reproductive health [39].

## 4. Mechanisms Involved in Bisphenol F-Induced SLD

The development of SLD under BPF exposure appears to be driven predominantly by PXR-independent mechanisms, with only limited and largely indirect support for a causal PXR-dependent contribution [7]. While BPF can modulate several nuclear receptors In vitro, most experimental evidence indicates that, unlike BPA, BPF does not act as a potent direct activator of human or mouse PXR isoforms [40,41]. Consequently, its steatogenic effects arise primarily through oxidative stress, ER dysfunction, mitochondrial impairment, PTMs, epigenetic remodelling, and direct disruption of lipid metabolism, all of which are pathways that are substantially independent of classical PXR signalling [6,8,42,43,44,45]. To address this dual axis concept and evidence hierarchy, the following subsections first outline the general PXR framework and summarize the weak evidence for direct BPF–PXR activation, then detail the better supported PXR-independent pathways.

### 4.1. The PXR-Dependent Axis: Conceptual Framework and Limited BPF Evidence

#### 4.1.1. PXR as a Master Metabolic Regulator—General Framework

PXR, also known as NR1I2, is a member of the nuclear receptor superfamily that plays a central role in regulating both endobiotic and xenobiotic metabolism [42]. It is highly expressed in the liver and intestines, which are organs vital for detoxification, drug metabolism, and chemical clearance [11]. PXR functions as a ligand-activated transcription factor, responding to a wide spectrum of structurally diverse compounds, including pharmaceuticals, herbal products, dietary constituents, environmental contaminants, and endogenous metabolites such as bile acids and steroid hormones [11,42].

Upon ligand binding, PXR undergoes conformational change and heterodimerizes with the retinoid X receptor (RXR), enabling the PXR–RXR complex to bind PXR response elements (PXREs) in target gene promoters and induce transcription of phase I (oxidative enzymes such as CYP3A4 and CYP2B6), phase II (conjugating enzymes such as UGT1A1), and phase III (efflux transporters such as ABCB1 P-glycoprotein) xenobiotic-metabolizing enzymes and transporters [11,46]. This orchestrated regulation maintains hepatic metabolic homeostasis and confers protection from chemical or xenobiotic stress [11,42]. Beyond detoxification, PXR integrates lipid, glucose, bile acid, and inflammatory signalling, and its activation can promote hepatic triglyceride (TG) accumulation via upregulation of CD36 and PPARγ. Persistent or excessive PXR activation can aggravate hepatic steatosis and steatohepatitis [11,47,48]. These pleiotropic functions make PXR mechanistically plausible as a mediator linking environmental chemical exposure to metabolic liver injury. However, the specific role of PXR in BPF-induced SLD remains uncertain and requires critical reassessment.

#### 4.1.2. BPF Interaction with PXR: Weak Direct Agonism

In vitro profiling indicates that BPF can interfere with nuclear receptor signalling, including PXR, and alter steroidogenic activity with potencies comparable to BPA [49]. In one of the QSAR models for cancer, where BPA was experimentally tested positive, BPF produced positive predictions [50]. However, several studies indicate that, unlike BPA, BPF does not act as a potent activator of human (hPXR) or mouse (mPXR) isoforms [40,41]. Specifically, Sui et al. [40] demonstrated that BPF exposure did not activate hPXR or mPXR, while Grimaldi et al. [41] reported absence of PXR activation in human cervical cancer cells treated with BPF. Structural analysis reveals that BPF lacks two critical methyl groups present in BPA’s bridge region, which are essential for maintaining hydrophobic contacts and hydrogen bonding interactions within the PXR ligand-binding domain [40] (Figure 1). This structural difference accounts for BPF’s markedly reduced binding affinity and weak or absent direct agonism.

Despite weak evidence of direct PXR activation in vitro, some in vivo studies reported marked activation of CYP3A, increased PPARγ, and hepatic TG accumulation that is believed to result from PXR activation, under BPF exposure [6,8,45]. This apparent paradox is more parsimoniously explained by indirect mechanisms rather than strong ligand-dependent PXR activation, e.g., via indirect PXR sensitization via oxidative stress and bile acid disturbances, cross-talk with other nuclear receptors, metabolic context-dependent PXR modulation and species and experimental system artefacts [47]. BPF-induced ROS and ER stress may subtly modulate PXR activity through perturbation of co-regulators, changes in RXR availability, or altered cellular redox balance, all of which are effects that do not require direct ligand binding [45,51]. BPF may also indirectly activate downstream PXR target genes through activation of overlapping nuclear receptors (e.g., CAR, PPARs, FXR) that share common promoter elements or co-regulators [42,52].

Meanwhile, PXR responsiveness is highly dependent on the cellular energy state. AMPK phosphorylation suppresses PXR activity, while mTOR activation enhances it [45]. Under BPF-induced oxidative stress and nutrient depletion, these PTMs may paradoxically suppress rather than enhance PXR activity, explaining discrepancies between high-dose cytotoxic versus low-dose subtle effects [45]. Transient reporter assays often lack essential co-activators (SRC-1, PGC-1α) required for robust PXR transcriptional activation in vivo. Moreover, species differences in the PXR ligand-binding domain (~76% homology between hPXR and mPXR) mean that murine models may not faithfully represent human PXR responsiveness to BPF [53].

Notably, no study has yet demonstrated that genetic deletion, knock-in, or pharmacological antagonism of PXR attenuates BPF-induced SLD. In contrast, PXR knockout or antagonism does reduce hepatic lipid accumulation and inflammation in certain xenobiotic-induced or diet-induced steatosis models. The absence of such causal validation in BPF models is a major limitation and suggests that any PXR involvement is secondary or epiphenomenal rather than driving.

### 4.2. PXR-Independent Mechanisms: Integrated Mechanistic Framework

In contrast to the equivocal PXR data, multiple independent in vitro and in vivo studies provide convergent evidence that BPF induces steatotic liver disease via PXR-independent mechanisms [4,6,8,12,33,43,44,45,54,55,56]. BPF-induced hepatotoxicity proceeds through a coordinated multi-pathway cascade in which oxidative stress and ER dysfunction act as primary initiating events that converge on mitochondrial dysfunction as a central mechanistic hub. Concurrent activation of innate immune pathways, dysregulation of PTMs, and epigenetic remodelling together drive persistent metabolic dysfunction and hepatic lipid accumulation, even in the absence of robust classical PXR signalling.

#### 4.2.1. Oxidative Stress and ER Dysfunction: Primary Initiating Events

Oxidative stress represents a foundational mechanism underlying BPF-induced hepatotoxicity, characterized by simultaneous elevation of reactive oxygen species (ROS) and reactive nitrogen species (RNS) in the setting of compromised antioxidant defences. BPF suppresses hepatic antioxidant enzyme activities (superoxide dismutase (SOD), catalase (CAT), glutathione peroxidase (GPx)) and depletes reduced glutathione (GSH), increasing the GSSG/GSH ratio and indicating heightened oxidative burden and impaired detoxification capacity [57]. Functionally, this promotes sustained lipid peroxidation, protein and DNA oxidation, and metabolic dysregulation that favours lipogenesis and systemic inflammation, thereby driving the progression of hepatic steatosis [58]. In parallel, redox-dependent PTMs directly modulate lipid metabolic signalling. For example, competitive cysteine oxidation and ubiquitination of Insig-2 can stabilize this ER-resident scaffold and restrain SREBP-driven cholesterol and lipid biosynthesis, illustrating how oxidative cues are integrated into lipid homeostatic control [59]. Framing BPF within this context suggests that oxidative stress does not merely damage macromolecules but also reprograms lipid signalling nodes such as the Insig–SREBP axis, strengthening the mechanistic link between BPF-induced redox imbalance, dyslipidemia, and progressive hepatic injury.

Developmental BPF exposure reveals heightened oxidative vulnerability during critical growth windows. Perinatal exposure in rats and gestational exposure in mice increase hepatic lipid peroxidation, compromise membrane integrity, suppress CAT and GSH activity, and downregulate antioxidant genes such as Sod1, Sod2, and Cat in the offspring of Long Evans rats and ICR mice, respectively [12,44]. These persistent alterations in antioxidant capacity promote steatogenesis and indicate that early BPF exposure establishes enduring oxidative vulnerability that can persist into adulthood, mechanistically linking perinatal toxicant exposure to later-life metabolic liver disease [12,44].

In vitro data across multiple species confirm that BPF-induced oxidative injury is a fundamental cellular response rather than a species-specific artefact. Rainbow trout (*Oncorhynchus mykiss*) hepatocytes exposed to BPF exhibit reductions in CAT activity, GSH depletion, elevated lipid peroxidation, and increased malondialdehyde (MDA) levels, while human hepatocytes and mouse liver show similar increases in MDA after BPF exposure [43]. An increase in MDA production has also been reported in human hepatocytes (L02) and the livers of male C57BL/6J mice following BPF exposure for 24 h and 30 consecutive days, respectively [6]. In parallel, BPF-induced ER dysfunction activates the unfolded protein response (UPR), with upregulation of HSPA5/GRP78, ATF6, and DDIT3/CHOP and enrichment of bZIP transcription factor motifs (ATF4, XBP1, CHOP) in chromatin-accessibility datasets [8]. The three canonical UPR branches—PERK–eIF2α–ATF4–CHOP, IRE1–XBP1, and ATF6—collectively couple chronic ER overload to TG accumulation and hepatocyte apoptosis, creating a coordinated programme that attempts to restore ER proteostasis but simultaneously promotes lipid accumulation and death signalling [60].

Oxidative and ER stress then engage in a bidirectional amplification loop that progressively overwhelms adaptive capacity. ROS disrupts ER calcium homeostasis by oxidatively modifying calcium-handling proteins and ER chaperones, leading to dysregulated calcium signalling, impaired protein folding, and protein aggregation [61]. Conversely, ER stress amplifies oxidative stress via PERK–eIF2α–ATF4–CHOP-driven induction of pro-oxidant enzymes and suppression of antioxidant genes, increasing net ROS production. Dysregulated calcium signalling enhances mitochondrial calcium uptake, promoting mitochondrial ROS generation and establishing a critical mechanistic link between ER dysfunction, oxidative injury, and mitochondrial impairment, which in turn feeds forward into inflammatory activation and steatotic progression [62].

#### 4.2.2. Mitochondrial Dysfunction and Energy Imbalance: A Central Hub

Mitochondrial dysfunction is a hallmark of BPF-induced steatotic liver disease and serves as a key convergence point for oxidative stress, ER stress, and metabolic dysregulation. Excess ROS damages electron transport chain (ETC) complexes, perturbs mitochondrial membrane potential, and reduces ATP production, compromising hepatic bioenergetics while paradoxically increasing electron leakage and further ROS generation [63,64]. Structural abnormalities such as mtDNA damage, cristae disorganization, and membrane rupture occur alongside activation of intrinsic apoptotic pathways (mitochondrial outer membrane permeabilization, cytochrome c release, caspase activation), progressively reducing hepatocyte viability during sustained BPF exposure [65].

Beyond passive ROS-mediated damage, BPF actively disrupts mitochondrial dynamics via aberrant activation of Drp1), a key regulator of mitochondrial fission. Upon BPF exposure, Drp1 is recruited to the mitochondrial outer membrane, where its GTPase-dependent oligomerization drives excessive mitochondrial fragmentation [6]. This Drp1-driven fission results in fragmented, dysfunctional mitochondria with impaired oxidative phosphorylation, enhanced electron leakage, diminished ATP synthesis, and increased ROS generation at multiple ETC sites, which collectively promote lipid droplet accumulation and non-alcoholic fatty liver disease (NAFLD)-like phenotypes in vivo [6]. Functional studies using Drp1 inhibition or genetic knockdown manage to restore mitochondrial integrity and oxidative phosphorylation and markedly attenuate hepatic lipid deposition, providing strong evidence that Drp1-mediated fission is causally involved in BPF-induced hepatotoxicity [6].

The dysfunctional mitochondrial network generated by ROS damage and Drp1-mediated fragmentation establishes a self-amplifying pathological cycle. Persistent ROS production further oxidizes respiratory chain proteins and inner mitochondrial membrane lipids, while structural disruption further compromises ETC efficiency and tight electron control, thereby sustaining oxidative injury [66]. This leads to a profound energy crisis as ATP levels fall and membrane potential collapses, reprogramming hepatocellular metabolism away from fatty acid oxidation toward lipid synthesis [67]. Three interrelated processes drive this shift: ATP-dependent β-oxidation is curtailed because the CPT1 system and downstream oxidative steps cannot operate efficiently under energy depletion [68]; de novo lipogenesis is paradoxically activated via SREBP-1c and related pathways, promoting TG synthesis and cytoplasmic lipid droplet accumulation [69]; and autophagic lipid clearance is impaired because autophagosome formation, trafficking, and lysosomal function are ATP-intensive [70], leading to reduced autophagic flux and further lipid retention.

Integration of oxidative, ER, and mitochondrial stress underscores mitochondrial dysfunction as both a consequence and amplifier of BPF toxicity. ER stress–mediated calcium dysregulation drives mitochondrial calcium overload [71], impairing oxidative phosphorylation and further increasing mitochondrial ROS [72], while mitochondrial ROS feeds back to damage ER calcium-handling proteins and chaperones, perpetuating ER stress. This intertwined network explains why mitochondrial dysfunction is a critical convergence point through which early oxidative and ER insults are translated into overt steatotic liver disease. The demonstration that Drp1 inhibition can reverse BPF-induced hepatic lipid accumulation highlights mitochondrial dynamics as a therapeutically tractable node, particularly for intervening during the early steatotic phase before irreversible fibrotic remodelling occurs.

#### 4.2.3. Inflammation: Convergence of Innate Immune Activation and Hepatocyte Injury

BPF-induced oxidative and mitochondrial stress converge on robust activation of innate immune pathways, creating a self-perpetuating inflammatory microenvironment that amplifies hepatocyte injury and fibrogenesis [12,44]. Injured hepatocytes release damage-associated molecular patterns (DAMPs), including ATP, nucleic acids, and other danger signals, which activate Kupffer cells via toll-like receptors and NOD-like receptors. This DAMP–PRR interaction rapidly converts Kupffer cells from a quiescent, tissue-protective state into a pro-inflammatory phenotype [73].

Activated Kupffer cells secrete a broad array of pro-inflammatory cytokines (IL-1β, IL-6, TNF-α) and chemokines (CCL2, CCL5, CXCL10) that recruit neutrophils, monocytes, and other immune cells to the liver [55,74,75]. Infiltrating immune cells further intensify inflammation, increase ROS production via NADPH oxidase, and contribute to secondary hepatocyte injury, thereby sustaining chronic hepatic inflammation. Within this context, BPF directly engages two critical inflammatory signalling nodes: the NLRP3 inflammasome and the NF-κB transcriptional axis [44]. Excess ROS and cell damage activate the NLRP3 complex, promoting caspase-1 activation and maturation of IL-1β and IL-18, while ROS-dependent degradation of IκB permits NF-κB nuclear translocation and transcription of pro-inflammatory cytokines, chemokines, and adhesion molecules such as ICAM-1 and VCAM-1 [76,77].

The net effect is a bidirectional amplification loop between oxidative stress and inflammation. Elevated ROS activates NLRP3 and NF-κB, whereas NF-κB-driven transcription upregulates pro-oxidant enzymes (e.g., NOX2, iNOS) and downregulates antioxidant genes, thereby sustaining and intensifying ROS generation [78]. Meta-analytic data across environmental toxicants, including bisphenols, indicate a shift in the hepatic cytokine milieu toward higher IL-1β, TNF-α, and IL-6 with relative suppression of anti-inflammatory mediators such as IL-10 and TGF-β, locking the liver into a pathogenic inflammatory state [79]. This chronic inflammatory environment not only perpetuates steatohepatitis but also promotes hepatic stellate cell activation and fibrogenesis, embedding BPF-induced oxidative and mitochondrial stress within a broader immunopathological context that accelerates progression toward advanced metabolic liver disease [58,74].

#### 4.2.4. Post-Translational Modifications: Lipogenic Reprogramming Independent of PXR Activation

PTMs, such as phosphorylation, acetylation, ubiquitination, and SUMOylation provide a flexible regulatory layer through which BPF can reprogramme hepatic metabolism independent of nuclear receptor signalling [80]. Under physiological conditions, these modifications fine-tune protein function, stability, subcellular localisation, and protein–protein interactions. Conversely, under BPF exposure, aberrant activity or expression of PTM-modifying enzymes (kinases, phosphatases, acetyltransferases, deacetylases) alters the activity of key metabolic enzymes and transcription factors, driving disease-promoting metabolic reprogramming without requiring strong PXR activation [80,81].

A central PTM target in this context is SREBP-1c, a master regulator of lipogenic gene expression [45]. BPF exposure enhances phosphorylation-dependent nuclear translocation of SREBP-1c in hepatocytes and mouse liver, indicating activation through PTMs rather than simple transcriptional upregulation. Once in the nucleus, SREBP-1c binds sterol regulatory elements and recruits co-activator complexes to induce transcription of lipogenic enzymes such as ACC, FAS, and SCD1. Coordinated upregulation of these enzymes amplifies de novo fatty acid synthesis and enriches the pool of TG-forming unsaturated fatty acids, directly contributing to hepatic TG accumulation and steatosis [45].

BPF also dysregulates the AMPK–mTOR energy-sensing axis through reciprocal phosphorylation changes that favour anabolism [82]. Suppression of AMPK phosphorylation removes a key metabolic brake that normally inhibits ACC and mTOR signalling during energy stress, permitting lipogenesis to proceed despite oxidative stress and ATP depletion [45]. Simultaneously, enhanced phosphorylation of mTOR promotes SREBP-1c activation, upregulates ACC and FAS, and suppresses catabolic processes such as autophagy and fatty acid oxidation. These dual shifts, which are reduced pAMPK and increased p-mTOR, lock hepatocytes into an obligate anabolic state, providing a PTM-based explanation for how BPF drives hepatic lipid accumulation independent of classical PXR signalling. The reversible nature of phosphorylation suggests that targeting PTM pathways (e.g., AMPK activators, mTOR inhibitors) could offer effective therapeutic strategies against BPF-induced metabolic liver disease by resetting the energy rheostat and restoring balance between anabolism and catabolism [45,83].

#### 4.2.5. Epigenetic Modifications: Heritable Reprogramming and Metabolic Memory

Epigenetic modifications constitute a distinct mechanistic layer through which BPF can induce persistent hepatic dysfunction that outlasts active exposure. These modifications include DNA methylation, histone tail modifications, chromatin-remodelling events that alter DNA accessibility, and dysregulation of non-coding RNAs [8]. Unlike acute changes in ROS, mitochondrial function, or PTM state, epigenetic alterations can stabilize disease-associated transcriptional programmes over long-time scales and may even convey risk across generations [84,85].

BPF-induced epigenetic changes have substantial functional consequences for hepatic lipid metabolism. Aberrant DNA methylation—particularly altered methylation of CpG-rich promoter regions—can modulate transcription of key regulators such as PPARα, SREBP-1c, and FASN, disrupting the balance between lipid synthesis and oxidation and thereby promoting steatosis [54]. In parallel, BPF exposure alters expression of specific microRNAs (e.g., miR-122, miR-34a) and long non-coding RNAs, which modulate hepatocyte proliferation, apoptosis, lipid metabolism, and inflammatory signalling via post-transcriptional regulation of target mRNAs [85,86]. These non-coding RNA changes complement DNA methylation and chromatin remodelling to generate a multifaceted epigenetic programme that favours lipid accumulation and inflammatory activation [85].

Multi-omics analyses provide emerging evidence that BPF exposure reconfigures the hepatic epigenetic landscape in an integrated manner [8]. Transcriptomic, metabolomic, and ATAC-seq datasets indicate that BPF alters chromatin accessibility at loci enriched for bZIP transcription factor motifs (ATF4, XBP1, CHOP), linking ER stress responses with lipid metabolic networks [8]. These changes include promoter methylation shifts (e.g., at SREBF1), altered chromatin accessibility that modulates transcription factor binding, and non-coding RNA dysregulation, together establishing a stable “epigenetic memory” of exposure [8,12,87,88]. This memory helps explain why developmental BPF exposure can yield metabolic phenotypes in adult offspring: epigenetic marks laid down during critical developmental windows may persist into adulthood and possibly transmit susceptibility to subsequent generations, with significant implications for life-course and transgenerational risk assessment.

#### 4.2.6. Integration: Multi-Hit Mechanistic Model

The collective evidence across oxidative stress, ER dysfunction, mitochondrial impairment, inflammatory activation, PTM dysregulation, and epigenetic remodelling supports a multi-hit mechanistic model of BPF-induced steatotic liver disease that operates largely independent of PXR [6,8,42,43,44,45]. The primary insult involves early BPF-induced ROS generation and ER calcium dysregulation [8], which occur in parallel but quickly become interlinked through bidirectional amplification, depleting antioxidant reserves and perturbing ER proteostasis [43]. During the amplification phase, oxidative stress impairs ER protein folding, ER stress–driven calcium leakage fuels mitochondrial ROS production, and DAMP release initiates inflammatory signalling via PRRs and inflammasomes, gradually exhausting adaptive responses and converting the UPR from protective to pro-apoptotic [60,73].

As these stresses persist, hepatocytes transition to a commitment phase in which mitochondrial dysfunction and inflammatory signalling become self-sustaining. Drp1-mediated mitochondrial fission and AMPK–mTOR axis dysregulation reprogramme cellular metabolism toward lipogenesis, while NF-κB and NLRP3 activation establish a chronic inflammatory milieu that exacerbates oxidative injury and promotes stellate cell activation and fibrogenesis [6,44,45]. Epigenetic alterations then consolidate this pathological state by stabilizing disease-promoting gene expression patterns, creating metabolic memory that maintains susceptibility even after BPF exposure has ceased [8,87].

This integrated framework explains why antioxidant depletion and ER stress markers correlate closely with hepatic lipid accumulation and why interventions targeting single pathways often yield only partial benefit. Effective resolution of BPF-induced hepatotoxicity will likely require multi-target strategies that restore redox balance, ER proteostasis, mitochondrial integrity, and inflammatory homeostasis simultaneously [6,43,44]. In particular, therapeutic approaches aimed at convergence points, such as the AMPK–mTOR energy axis, Drp1-mediated mitochondrial dynamics, and inflammasome/NF-κB signalling, hold promise for achieving more robust and durable reversal of BPF-induced metabolic liver disease than monotherapies directed at isolated mechanisms.

## 5. BPF Induced Disruption of Lipid Metabolism

Chronic inflammation and mitochondrial impairment can result in the disruption of hepatic lipid metabolism. The production of cytokines during chronic inflammation alters the normal balance between lipid synthesis and degradation by boosting de novo lipogenesis and inhibiting fatty acid oxidation [89]. Meanwhile, mitochondrial dysfunction induced by toxins diminishes energy production and impairs the liver’s capacity to metabolize fatty acids efficiently [90]. Lipids that accumulate within hepatocytes will lead to lipotoxicity and steatosis. A detrimental cycle begins when the accumulation of harmful lipid intermediates worsens inflammation and cell damage [91]. Prolonged inflammation and lipid dysregulation will activate hepatic stellate cells, resulting in fibrosis that may eventually progress to cirrhosis or HCC [92].

BPF interferes with hepatic lipid metabolism via various pathways that collectively facilitate excessive fat storage in hepatocytes [93]. BPF induces dysregulation of lipid metabolism by impairing lipid homeostasis, particularly in the liver and adipose tissue of high-fat-diet mice [33]. Sun et al. [33] treated mice with 0.05 mg/kg body weight of BPF for 8 weeks, while a second group received 5 mg/kg body weight of BPF during the first week, followed by 0.05 mg/kg body weight of BPF for 7 weeks, which was combined with a high-fat diet. They found that the hepatic lipid metabolism was disrupted, as evidenced by reductions in TGs and cholesterol levels, as well as significant changes in fatty acid composition, with increased n-6 fatty acids, while n-3 fatty acids were decreased. Analysis of fatty acid desaturation showed alterations in the Δ5/Δ6-desaturase ratios, implying modulated lipid remodelling in response to combined metabolic and dietary stress. These data indicate that BPF exposures, especially against the backdrop of a high-fat diet, can disrupt both the balance and biochemical pathways of lipid metabolism, thus contributing to hepatic lipid disturbance.

Other studies indicate that BPF exposure correlates with SLD, affecting lipid metabolites such as phospholipids, sphingolipids, and glycerides in murine livers. BPF significantly impacts the glycerophospholipid metabolic pathway, leading to disruptions in lipid metabolism [4,93]. In an in vivo experiment, C57BL/6J mice (7 weeks old) were administered BPF at doses of 0.00, 0.04, 0.40, and 4.00 mg/kg body weight for 30 consecutive days [93]. Histological examination confirmed a concentration-dependent increase in hepatic lipid droplet formation and lipid deposition, indicating the onset of steatotic changes. Supporting human data show higher BPF concentrations in individuals with moderate to severe NAFLD compared with healthy controls, correlating with elevated serum TG levels [93]. These findings from Wang et al. [93] and others collectively demonstrate that chronic low- to mid-dose of BPF exposure disrupts lipid homeostasis, remodels hepatic lipid architecture, and contributes to lipid droplet accumulation, underscoring BPF’s potential role in the development of MASLD and NAFLD-like conditions in humans.

BPF disrupts hepatic lipid balance by impairing the SIRT1–PPARα-autophagy pathway, leading to reduced lipid degradation and increased TG accumulation in both HepG2 cells and mouse liver [54]. Gene expression changes involving increased levels of multiple autophagy-related genes (ATG5, ATG7, ATG14, Beclin-1, LC3, p62) but reduced expression of SIRT1, LAMP1, TFEB, PPARα, and ATGL after BPF exposure reflect defective autophagic clearance and weakened fatty acid oxidation, resulting in marked lipid droplet buildup and steatotic changes. Thus, BPF-induced autophagy and mitochondrial dysfunction are central to early metabolic liver injury [54]. Male C57BL/6 mice (8 weeks old) exposed in vivo to 50 mg/kg/day of BPF for 30 days, and HepG2 cells treated in vitro with 10 μM of BPF, exhibited increased TGs and elevated fatty acids including linoleic, arachidonic, and palmitic acids. Mechanistically, BPF exposure activates SREBP-1c and PPARγ, enhances fatty acid elongation, and disrupts hepatic lipid homeostasis, ultimately promoting TG deposition and steatosis [4]. Table 1 shows summary evidence of BPF-induced SLD with proposed PXR-independent mechanisms.

## 6. Discussion

This review establishes that BPF-induced SLD develops through a coordinated PXR-independent multi-pathway cascade that mechanistically overlaps with MASLD whilst maintaining its identity as an environmentally triggered condition. This integrated framework distinguishes BPF-induced SLD as a distinct disease requiring targeted prevention and therapeutic strategies. Table 2 shows the summarized comparative mechanisms of BPF-induced SLD.

### 6.1. The PXR Paradox and Its Implications

BPF demonstrates a fundamental paradox: it fails to directly activate PXR in vitro yet produces apparent PXR-like responses in vivo [40,41]. This is resolved by recognizing that indirect stress-mediated sensitization, rather than direct ligand binding, accounts for in vivo observations [33]. BPF functions as a metabolic stressor whose effects on nuclear receptor networks are secondary consequences of cellular dysfunction, not as a classical xenobiotic receptor activation.

This has critical regulatory implications. Current xenobiotic screening paradigms relying on nuclear receptor assays would classify BPF as weak or inactive, systematically underestimating its hepatotoxic potential [94]. This disconnect highlights a fundamental limitation in environmental toxicology: compounds acting through stress pathways rather than receptor-mediated mechanisms escape conventional detection. Regulatory frameworks should therefore incorporate stress pathway biomarkers, e.g., oxidative stress indicators, mitochondrial function assays and inflammatory markers, alongside traditional receptor endpoints [45]. The experimental heterogeneity across BPF-PXR studies reflects broader methodological challenges, i.e., species differences in receptor architecture, concentration-dependent mechanistic switching, and absent co-regulators in reporter systems prevent any single approach from definitively characterizing toxicant-receptor interactions. Future studies should employ integrated approaches combining reporter assays, primary hepatocytes, humanized models, and genetic perturbation for triangulation.

### 6.2. Evidence Hierarchy: Well-Supported Versus Speculative Mechanisms

Strong evidence supported by replication across laboratories, multiple species, and functional validation includes oxidative stress, Drp1-mediated mitochondrial fission, and NLRP3/NF-κB inflammatory activation [6,12,33,43,44]. Notably, Drp1 inhibition reverses BPF-induced hepatic lipid accumulation, establishing causality rather than mere correlation [6]. In contrast, moderate evidence mechanisms, namely PTM dysregulation (AMPK-mTOR axis) and epigenetic modifications, are mechanistically compelling but derive from limited studies lacking functional validation through kinase inhibitors/activators and epigenetic editors [8,45,87]. Most critically, direct PXR involvement remains supported only by weak evidence with absent causal validation. Despite plausibility, no loss-of-function studies demonstrate that PXR knockout, knockdown, or antagonism attenuates BPF-induced SLD. This evidentiary gap should curtail attributing BPF effects to PXR-dependent mechanisms. Therapeutic development should therefore prioritize validated stress pathways over PXR or incompletely validated PTM nodes.

**Table 2:** The summarized mechanisms of BPF-induced SLD.

Mechanisms	PXR Dependence	Evidence Strength	In Vitro OR In Vivo Studies	Major Limitations	Functional Validation	References
Oxidative stress and ROS elevation	None (direct)	Strong	Both robust	Non-physiological BPF concentrations (in vitro); limited time-course studies; unclear dose–response relationship for environmentally relevant exposures	Antioxidant enzyme activity measured; GSH/GSSG ratios quantified; MDA/lipid peroxidation confirmed	Zhang et al. [6], Meng et al. [12], Aykut et al. [43], Linillos-Pradillo et al. [44], Lința et al. [63], Sangwan et al. [95], Charles & Prince [96]
ER stress and UPR activation	None (direct)	Strong	Both; strong multi-omics	Chromatin accessibility data largely correlative; limited mechanistic link between UPR branch activation and specific outcomes; no UPR component knockdown studies	Transcriptomic evidence for ATF6, PERK, IRE1 branch activation; multi-omics enrichment for ER-related transcription factors	Fan et al. [8], Luo et al. [60], Chipurupalli et al. [97]
Drp1-mediated mitochondrial fission	None (direct)	Strong	In vivo primarily	Limited to one key study; Drp1 inhibition only tested in one model; sex differences not examined; age-dependence unclear	Drp1 inhibition restores mitochondrial integrity and reduces lipid deposition	Zhang et al. [6]
NLRP3/NF-κB inflammatory activation	None (direct)	Strong	Both; consistent across models	Mostly descriptive (cytokine measurement); limited mechanistic dissection of NLRP3 vs. NF-κB contributions; inflammasome component knockdown not tested; microbiota role speculative	Cytokine elevation replicated; NLRP3 activation	Zhang et al. [6], Linillos-Pradillo et al. [44], Taru et al. [98], Jee et al. [73], Peinado et al. [76], Marques et al. [77]
SREBP-1c PTM and lipogenic reprogramming	None (direct)	Moderate	Both (limited In vivo)	Only one comprehensive PTM study (Xue et al.); limited mechanistic detail on which PTMs activate SREBP-1c; no SREBP-1c knockout rescue; unclear whether PTMs are primary drivers or consequences	SREBP-1c nuclear translocation and lipogenic enzyme upregulation demonstrated	Cao et al. [99]
AMPK–mTOR dysregulation	None (direct)	Moderate	Both (limited in vivo)	Phosphorylation status measured but causality not tested (no pAMPK/mTOR manipulation); relationship to energy deficit unclear; tissue-specific effects not examined	pAMPK decrease and p-mTOR increase replicated; but no pharmacological/genetic intervention	Xue et al. [45], Yang et al. [83]
Autophagy disruption and SIRT1–PPARα impairment	None (direct)	Moderate	Both (limited in vivo)	Single study; no ATG gene knockout/knockdown; SIRT1/PPARα inhibition not tested; unclear whether autophagy impairment is primary or secondary to mitochondrial dysfunction	Autophagy marker changes (LC3, p62, ATG genes) documented; lipid droplet accumulation confirmed	Wang et al. [54]
Epigenetic remodelling (DNA methylation, chromatin accessibility, miR dysregulation)	None (direct)	Moderate–weak	Both; limited in vivo	Correlative multi-omics data only; no CRISPR-based epigenetic editing to test causality; no pharmacologic epigenetic modifier studies; transgenerational effects entirely speculative; unclear whether epigenetic changes are drivers or consequences of stress	Chromatin accessibility and transcriptomic changes documented; no functional validation of epigenetic causality	Fan et al. [8], Meng et al. [12], Ramirez et al. [87], Merrel et al. [100]
Indirect PXR modulation via oxidative/metabolic stress	Indirect (non-ligand-dependent)	Weak/speculative	Primarily in vivo	No direct evidence that PXR knockout/antagonism attenuates BPF-induced SLD; In vitro PXR activation assays consistently negative; mechanistic link between oxidative stress and PXR modulation not demonstrated; species-specific differences unexplained; no humanized PXR model tested	CYP3A and PPARγ induction observed in vivo, but causality to PXR unproven; could reflect CAR or PPAR-driven responses	Sun et al. [33], De Battistis et al. [52], Xue et al. [45], Niu et al. [51]
Direct PXR ligand-dependent activation	Direct (ligand- dependent)	Not supported	In vitro only (negative findings)	Multiple independent In vitro studies show NO hPXR or mPXR activation by BPF; structural differences (loss of methyl groups) explain reduced affinity; no in vivo validation of ligand-dependent mechanism; contradicts weak PXR binding and agonism data	Transient transfection reporter assays negative for BPF; structural model predicts poor binding	Sui Et Al. [40], Grimaldi Et Al. [41]

Abbreviations: ROS: Reactive Oxygen Species; BPF: bisphenol F; MDA: malondialdehyde; ATF6: activating transcription factor 6; GSH: glutathione; GSSG: Glutathione Disulfide; NLRP3: NLR family pyrin domain containing 3; Drp1: Dynamin-Related Protein 1; PPARα: Peroxisome proliferator-activated receptor alpha; ACC: Acetyl-CoA Carboxylase; SREBP-1c: Sterol Regulatory Element-Binding Protein-1c; PPARγ: Peroxisome Proliferator-Activated Receptor Gamma; AMPK: AMP-activated protein kinase; p-mTOR: phosphorylated mTOR; UPR: Unfolded Protein Response; ER: Endoplasmic Reticulum; PERK: Protein kinase R-like endoplasmic reticulum kinase; IRE1: inositol-requiring enzyme 1; NF-κB: Nuclear factor kappa-light-chain-enhancer of activated B cells; PTM: Post-Translational Modifications; PXR: Pregnane-X-Receptor; CYP3A: Cytochrome P_450_ 3A; CAR: Constitutive Androstane Receptor; SLD: Steatosis Liver Disease; LC3: Microtubule-associated protein light chain 3; p62: sequestosome 1; ATG: Autophagy Related; SIRT1: sirtuin 1.

### 6.3. Why PXR-Independent Pathways Dominate

Three mechanistic principles explain PXR-independent pathway dominance with implications extending beyond BPF. First, a potency differential exists whereby PXR-independent mechanisms operate through direct molecular perturbation (ROS generation, mitochondrial fission), generating rapid, potent responses, whilst PXR involvement requires indirect sensitization that is inherently weaker [6]. Second, positive feedback amplification creates self-reinforcing bidirectional loops (oxidative stress ↔ ER stress, ROS ↔ inflammation, mitochondrial dysfunction ↔ metabolic dysregulation) that overwhelm secondary PXR contributions, which lack intrinsic positive feedback and are negatively regulated under stress [64]. Third, temporal dynamics favour stress pathways operating within minutes to hours; PXR-dependent transcription requires hours to days, meaning cellular dysfunction becomes established before compensatory PXR responses develop [94]. These principles predict that stress pathway interventions (antioxidants, mitochondrial protectants, anti-inflammatory agents) will exceed PXR modulation in effectiveness, and that early intervention before amplification becomes self-sustaining will be therapeutically critical. These principles likely also apply broadly to environmental toxicants causing cellular stress through ROS, mitochondrial dysfunction, or inflammatory activation.

### 6.4. Critical Knowledge Gaps and Research Priorities

The most critical gap is the absence of causal validation for PXR involvement. Definitive PXR causality validation using PXR knockout hepatocytes, whole-animal models, and humanized transgenic mice would resolve the mechanistic controversy and guide therapeutic targeting [101]. Equally important is the near-complete absence of human data, whilst the mechanistic framework is derived from rodent and in vitro studies. Whether these mechanisms operate in human liver tissue at environmentally relevant BPF exposures remains unknown. Longitudinal cohort studies measuring BPF biomarkers alongside hepatic function markers would establish human relevance and identify dose–response relationships, and occupational studies of elevated-exposure workers (e.g., thermal paper handlers, plastics manufacturers) could provide natural experiments for assessing human hepatotoxicity [79]. Additional high-priority gaps include incomplete dose–response characterization spanning environmentally relevant to toxicological doses, unvalidated PTM causality through activator/inhibitor studies, untested transgenerational epigenetic inheritance across F1-F3 generations, limited sex-stratified data on sexual dimorphism mechanisms, and absent therapeutic validation beyond partial Drp1 inhibition studies. Therefore, a coordinated research programme should address critical priorities (PXR causality, humanized systems), high priorities (Dose–Response, transgenerational epigenetics, biomarker development), and translational priorities (human epidemiology, therapeutic intervention trials, microbiota–hepatic axis characterization through germ-free experiments).

### 6.5. Regulatory and Public Health Implications

Current regulatory screening emphasizing nuclear receptor activation would classify BPF as low risk based on weak PXR activation, yet evidence demonstrates substantial hepatotoxic potential through stress pathways [6,8,12,43,44]. Thus, regulatory frameworks must expand to incorporate stress pathway biomarkers alongside receptor endpoints. The structure-activity relationship carries particular significance: if PXR agonist potency follows BPA > BPF > BPS whilst oxidative hepatotoxicity follows an inverse or equivalent pattern, regulatory replacement of BPA with “safer” analogues may not reduce hepatotoxic risk (Table 3). This suggests that environmental replacement of BPA with incompletely characterized analogues may inadvertently maintain or increase population health risk through “regrettable substitution” patterns. Comprehensive toxicological assessment, including stress pathway evaluation, is essential before regulatory approval of chemical substitutes. The developmental implications are equally significant, as evidence that perinatal BPF exposure establishes persistent epigenetic and metabolic vulnerabilities suggests that early-life exposure may heighten life-course disease risk even without continued adult exposure [6,8,87]. If transgenerational epigenetic inheritance is confirmed experimentally, current-generation BPF exposure could influence disease risk in subsequent generations, amplifying the public health significance of exposure prevention and suggesting that protecting pregnant women and young children from BPF exposure may be disproportionately important relative to adult exposure reduction. A comparative summary of BPA, BPF, and BPS binding affinity and ability to activate hPXR and mPXR is presented in Table 3.

### 6.6. Therapeutic Implications

**Figure 2:**
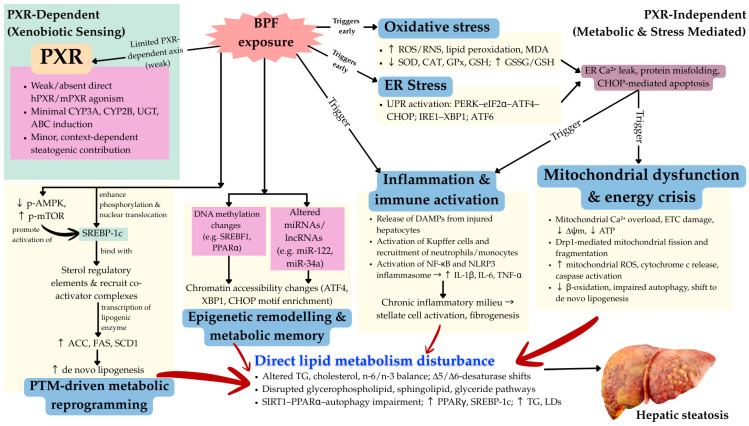
Integrated multi-hit model of BPF-induced SLD. BPF exposure leads to only weak, context-dependent activation of PXR, providing limited contribution to steatogenesis, while the predominant toxic actions proceed via PXR-independent pathways. In hepatocytes, BPF triggers early oxidative stress and endoplasmic reticulum (ER) stress, depleting antioxidant defences and activating unfolded protein response branches, which in turn disrupt calcium homeostasis and promote protein misfolding. These events converge on mitochondrial dysfunction characterized by Drp1-mediated mitochondrial fission, impaired oxidative phosphorylation, excessive mitochondrial reactive oxygen species, and an ATP deficit that suppresses β-oxidation and autophagy while favouring de novo lipogenesis. Parallel activation of inflammatory pathways, including NF-κB and NLRP3 inflammasome signalling in Kupffer cells and recruited immune cells, establishes a chronic pro-inflammatory microenvironment that amplifies hepatocyte injury and stimulates stellate cell activation and fibrogenesis. In addition, dysregulated post-translational modifications (e.g., SREBP-1c activation, reduced AMPK and increased mTOR signalling) and epigenetic alterations (DNA methylation, non-coding RNA changes, and chromatin accessibility reprogramming) reinforce lipogenic, pro-oxidant, and pro-inflammatory gene expression programmes. These molecular events converge on disruption of hepatic lipid metabolism, including impaired SIRT1–PPARα–autophagy signalling, altered fatty acid profiles, and glycerophospholipid remodelling, culminating in triglyceride accumulation, lipid droplet deposition, steatohepatitis, and progression toward fibrosis and advanced metabolic-associated steatotic liver disease.

Drp1-mediated mitochondrial fission represents the most validated therapeutic target, with functional evidence demonstrating that Drp1 inhibition reverses BPF-induced hepatic lipid accumulation. Thus, Drp1 inhibitors (Mdivi-1, P110) warrant investigation in preclinical therapeutic trials with attention to optimal timing and combination strategies [102]. The NLRP3 inflammasome and NF-κB axis represent well-validated inflammatory targets, with multiple approved or investigational agents (anakinra, canakinumab for IL-1β; various NF-κB inhibitors) available for repurposing studies [103]. The AMPK-mTOR energy axis offers theoretical therapeutic potential, with AMPK activators (metformin) and mTOR inhibitors (rapamycin, everolimus) representing candidates for investigation, though functional validation in BPF models remains necessary before clinical translation [104]. A key insight from the multi-hit mechanistic model is that single-pathway interventions will produce incomplete benefit due to the interconnected, self-amplifying nature of BPF-induced pathology, suggesting that combination strategies simultaneously targeting oxidative stress (antioxidants), mitochondrial dynamics (Drp1 inhibitors), and inflammation (NLRP3/NF-κB inhibitors) may be necessary to achieve durable disease reversal. Future therapeutic development should therefore prioritize combination approaches and identify optimal multi-target regimens through systematic preclinical evaluation. Figure 2 shows the proposed mechanism of BPF-induced SLD.

**Table 3:** The BPF-induced SLD via oxidative stress, mitochondrial dysfunction, inflammation, post-translational regulations, epigenetics and lipid disturbance.

Bisphenols	PXR Activation (In Vitro/In Vivo)	Structural Determinants	Key Findings	Potential Indirect Mechanisms	Authors
Bisphenol A (BPA)	Strong activator of hPXR; No effect on mPXR	Two methyl groups on bridge; para-hydroxy phenyl rings	Induces CYP3A4, CD36, PPARγ; triggers steatosis and hyperlipidemia	Direct ligand binding; Energetically stable direct ligand binding (LBD) fit	Sui Et Al. [40,105]
Bisphenol F (BPF)	Weak or no activation of hPXR/mPXR	Missing methyl groups; reduced hydrophobic complementarity	Mimics downstream PXR responses (CYP, lipogenic gene upregulation); causes SLD phenotypes	Indirect activation via oxidative stress, FXR–PXR crosstalk and CAR pathways	Ji et al. [24]; Han et al. [106]; Grimaldi Et Al. [55]
Bisphenol S (BPS)	No activation of hPXR or mPXR	Sulfone linker (SO_2_) disrupts hydrogen bond with Ser247	Weaker lipid-accumulating effect in rodent models	Alters AhR/ER signalling; no PXR dependence	Fang et al. [107]; Han et al. [106]

## 7. Conclusions

This review establishes that BPF-induced SLD operates predominantly through PXR-independent mechanisms, resolving the “PXR paradox” by showing toxicity initiates via oxidative stress and ER dysfunction, converging on mitochondrial impairment as a hub that drives self-amplifying loops of inflammation, post-translational dysregulation, and epigenetic remodelling to establish hepatic steatosis, with PXR involvement proven secondary and non-causal by absent loss-of-function validation. Replacing BPA with BPF risks perpetuating hepatotoxicity under current receptor-centric regulations that misclassify it as low risk, necessitating expanded screening with oxidative, mitochondrial, and inflammatory biomarkers, plus enhanced protection for pregnant women and children given perinatal exposure’s persistent risks. Future research should adopt integrative methodologies combining in vitro, in vivo, and computational models to elucidate the mechanistic role of BPF in PXR signalling and hepatic pathology, with priorities including definitive PXR causality validation, clarification of species-specific differences, dose–response relationships, and interactions with other nuclear receptors. Such approaches should be complemented by human epidemiological studies linking BPF biomarkers to liver injury, transgenerational investigations, and the development of new biomarkers related to PXR pathway activation to improve toxicological risk assessment. In parallel, therapeutic studies should focus on validated convergence points such as Drp1-mediated mitochondrial dynamics, NLRP3/NFκB-driven inflammation, and AMPK mTOR energy sensing, using multi-target strategies that can be applied more broadly to environmentally induced metabolic liver disease.

## Figures and Tables

**Table 1:** The proposed key mechanisms of BPF-induced SLD via PXR-independent.

Concentration of BPF	Duration of Exposure	Animal/Cells	Key Findings	Proposed Key Mechanisms	Authors
0, 15.63, 31.25, 62.50, 125, 250, and 500 µM	24 h	Hepatocytes isolated from the liver of rainbow trout (*Oncorhynchus mykiss*) (ex vivo)	↑ MDA: 15.63–250 µM ↑ SOD: 15.63–500 µM ↓ CAT: 15.63–500 µM ↑ GSH: 15.63; ↓ 500 µM GST: no changes ↑ GPx: 31.25–500 µM	Oxidative stress induction due to excessive ROS and LPO production; imbalance in antioxidant defences (SOD, CAT, GPx) leads to hepatocellular oxidative injury and lipid peroxidation.	Aykut & Kaptanur [43]
BPF-low-dose (LBPF) 0.0365 mg/kg b.w./day, and BPF-high-dose (HBPF) 3.65 mg/kg b.w./day	Diet (Total exposure days: 53 days) Premating— 14 days Mating— 10 days Pregnant— 23 days PND—6 days	Thirty-six female (eight weeks of age) and eighteen male (ten weeks of age) Long Evans rats (offspring) Perinatal Exposure	LBPF ↓: CAT, SOD, GPx, GRx, GST, GSH; ↑ GSSG, GSSG/GSH ratio, MDA, LPO ↑ Activation of NLRP3 inflammasome components (NLRP3, PyCARD, CASP1), ↑ pro-inflammatory cytokines (IL-1β, IL-18, IFN-γ, and TNF-α)	NLRP3 inflammasome activation driven by oxidative stress and ER stress; excessive ROS and lipid peroxidation trigger pro-inflammatory cytokine production (IL-1β, TNF-α), promoting steatohepatitis.	Linillos-Pradillo et al. [44]
In vitro: 10 µM In vivo: 0.05, 0.2 and 0.5 mg/kg	In vitro: 24 h In vivo: 30 consecutive days via gavage.	In vitro: human hepatocytes (L02) In vivo: Male C57BL/6J mice, aged 5–6 weeks (n = 6)(young adult)	Both L02 and mouse liver— ↑ MDA ↑ Drp1 ↓ mitochondrial ATP production capacity Lipid gene metabolism (↓ Acox1, PMP70, Pex5, Pex19) Mitochondrial-related lipid metabolism (↓ PPARα, CPT1α, ACSL1, Sod2, ↑ CoxIV) Promote aberrant mitochondrial fission and fusion Mouse liver: Accumulation of lipid droplets in liver	Mitochondrial-peroxisomal dysfunction via PPARα/CPT1α downregulation, resulting in disrupted β-oxidation, ATP depletion, and aberrant fission–fusion cycles (↑ Drp1) leading to lipid accumulation.	Zhang et al. [6]
100 ng/g bw/day	7th day of pregnancy to the 21st day after delivery.	Primigravida pregnant ICR mice (n = 8) (male mouse offspring)	↓ GSH, CAT ↓ mRNA gene expression (Sod1, Sod2, Cat) Weak activator of the Fxr-Shp pathway	Antioxidative enzyme suppression and impaired FXR-SHP signalling, diminishing bile acid regulation and mitochondrial defence against oxidative insults, promoting steatogenesis.	Meng et al. [12]
In vivo: 50 mg/kg/day In vitro: 10 µM	30 days via subcutaneous injection	In vivo: Male C57BL6 mice (8 weeks old) In vitro: HepG2 cells	↑ TG ↑ Fatty acids (9,12-Octadecadienoic acid, arachidonic acid, Dodecanoic acid, Eicosapentaenoic acid, Heptadecanoic acid, Palmitelaidic acid, Palmitic acid, α-Linolenic acid	Lipogenesis activation involving SREBP-1c/PPARγ upregulation and fatty acid elongation; disruption of lipid homeostasis, promoting triglyceride accumulation and hepatic steatosis.	Wang et al. [4]
0.05, 5 mg/kg bw	0.05 mg/kg bw BPF for 8 weeks 5 mg/kg bw BPF for 1st week; 0.05 mg/kg bw BPF for 7 weeks under HFD	HFD mice	↑ SOD, ↓ MDA Lipid metabolism disturbance: ↓ TGs, Cholesterol Changes in fatty acid metabolism: ↑ N-6 FAs, ↓ N-3 FAs	Disruption of fatty acid metabolism and desaturation ratio (Δ5/Δ6-desaturases) changes; demonstrates modulation of lipid remodelling and oxidative enzyme activity under combined metabolic stress.	Sun et al. [33]
0.5, 5, 50 µg/L	embryonic stage for 180 days	Wild-type (AB) zebrafish	5, 50 µg/L hepatic fibrosis and steatosis BPF shift microbiome composition in intestinal	Gut–liver axis modulation—altered bile acid metabolism and microbiota composition promoting endotoxin-driven hepatic inflammation and fibrosis.	Wang et al. [55]
0.00, 0.04, 0.40, and 4.00 mg/kg	30 consecutive days	C57BL/6J mice (7 weeks old) NAFLD in volunteers and patients with NAFLD	BPF ↑ lipid droplet and deposition in mouse liver (histology) ↑ TG: 0.04, 0.40, and 4.00 mg/kg ↓ glycerides: 4.00 mg/kg ↓ Sterol esters: 0.04, 4.00 mg/kg ↓ Phospholipids: 4.00 mg/kg ↓ Sphingolipids: 0.04, 0.40, and 4.00 mg/kg ↓ Fatty acyl: 4.00 mg/kg ↑ [BPF] in moderate to severe fatty liver ↑ TG	Lipidomic reprogramming—disturbance in glycerophospholipid and sphingolipid metabolism, impairing membrane integrity and signalling pathways that regulate hepatic lipogenesis.	Wang et al. [54]
In vitro: 10 μmol/L In vivo: 200 μg/kg body weight	In vitro: 24 h In vivo: 30 consecutive days via gavage	In vitro: HepG2 cells In vivo: C57BL/6J mice (male, 5 to 6 weeks old)	In vitro: lipid droplet accumulation increased and enlarged lipid droplets in HepG2 cells ↑ TGs, ↓ Cholesterol mRNA and protein levels-upregulated in ACC, FAS, SREBP-1c, CEBPA, PPARγ, SCD1, and AGPAT1 (lipogenesis), down-regulated DLK1 (anti-lipogenesis) ↓ phosphorylation of AMPK, ↑ p-mTOR In vivo: mRNA and protein levels-upregulated in ACC, FAS, SREBP-1c, C/EBPα, PPARγ, and SCD1 (lipogenesis) ↓ phosphorylation of AMPK, ↑ p-mTOR lipid droplet accumulation ↑ TGs, ↓ Cholesterol	Pro-lipogenic signalling via AMPK/mTOR suppression; enhanced SREBP-1c and FAS transcription promoting lipid accumulation; disrupted DLK1-mediated inhibitory feedback in lipogenesis.	Xue et al. [45]
In vitro: 100 μM In vivo: low dose (100 μg/kg bw/day) high dose (1000 μg/kg bw/day)	In vitro: 24 h In vivo: Three months	In vitro: AML12 cell line In vivo: Eight-week-old C57BL6 male mice	In vitro: mRNA expression levels of markers of lipid metabolism (upregulation Acot13, Cpt1a, Acox1, Acox2, Atf3) and ER stress (upregulation Atf6, Ern1, Eif2a and downregulation of Ddit3, Atf4, Cebpb). In vivo: Metabolomics findings (Reprogram the chromatin accessibility and enhancer landscape in the liver)	Epigenetic remodelling of lipid metabolism genes through altered histone acetylation and enhancer accessibility; persistent transcriptional reprogramming linked to hepatic lipid imbalance via ER stress	Fan et al. [8]
In vitro: 10 μM In vivo: 200 μg/kg/day	In vitro: 24 h In vivo: oral gavage daily for a consecutive 30-day period	In vitro: HepG2 In vivo: Male C57BL/6 mice (5–6 weeks old)	In vitro: Lipid droplet accumulates in hepG2 cells ↑ TG content mRNA levels of autophagy-related genes, SIRT1 and PPARα in HepG2 cells (↑ ATG5, ATG7, ATG14, Beclin-1, LC3, p62, Beclin-1; ↓ SIRT1, LAMP1, TFEB, PPARα, ATGL) In vivo: Lipid droplet accumulates in hepG2 cells ↑ TG content mRNA levels of autophagy-related genes (↓ SIRT1, LAMP1, PPARα, ATGL; ↑ p62, Beclin-1, ATG5, LC3)	Autophagy dysregulation—impaired SIRT1–PPARα–autophagy pathway reduces lipid degradation capacity; increased LC3/p62 accumulation signals defective autophagic clearance driving steatosis.	Wang et al. [87]

Abbreviations: ↑: increase; ↓: decrease; BPF: bisphenol F; MDA: malondialdehyde; SOD: superoxide dismutase; CAT: catalase; GSH: glutathione; GST: glutathione S transferase; GPx: glutathione peroxidase; GRx: glutathione reductase; µM: micro molar; h: hour; GSSG: Glutathione Disulfide; LPO: Lipid peroxidation; NLRP3: NLR family pyrin domain containing 3; PyCARD: PYRIN domain and a caspase-recruitment domain; CASP1: gene that codes for Caspase-1; IL-1β: Interleukin 1 beta; IL-18: Interleukin-18; IFN-γ: Interferon gamma; TNF-α: tumour necrosis factor alpha; Drp1: Dynamin-Related Protein 1; Acox1: acyl-CoA oxidase 1; PMP70: peroxisomal membrane protein 70; Pex5: Peroxisomal Biogenesis Factor 5; Pex19: Peroxisomal Biogenesis Factor 19; PPARα: Peroxisome proliferator-activated receptor alpha; CPT1α: Carnitine Palmitoyltransferase 1 alpha; ACSL1: Acyl-CoA Synthetase Long Chain Family Member 1; Sod2: Superoxide dismutase-2; CoxIV: Cytochrome c oxidase subunit IV; FXR: Farnesoid X Receptor; SHP: Small Heterodimer Partner; FA: fatty acid; TG: triglyceride; ACC: Acetyl-CoA Carboxylase; FAS: Fatty Acid Synthase; SREBP-1c: Sterol Regulatory Element-Binding Protein-1c; CEBPA: CCAAT-Enhancer-Binding Protein Alpha; SCD1: Stearoyl-CoA Desaturase 1; AGPAT1: 1-acylglycerol-3-phosphate O-acyltransferase 1; DLK1: Delta-like 1 homologue; AMPK: AMP-activated protein kinase; p-mTOR: phosphorylated mTOR.

## Data Availability

No new data were created or analyzed in this study.

## Abbreviations

The following abbreviations are used in this manuscript:

BPF	Bisphenol F
PXR	Pregnane-X Receptor
FXR	Farnesoid X Receptor
LXR	Liver X Receptor
CAR	Constitutive Androstane Receptor
CYP3A4	cytochrome P450 3A4
↑	Increase
↓	Decrease
PTMs	Post-Translational Modifications
MDA	Malondialdehyde
SOD	Superoxide dismutase
CAT	Catalase
GSH	Glutathione
GST	Glutathione S transferase
GPx	Glutathione peroxidase
GRx	Glutathione reductase
µM	Micro molar
h	Hour
GSSG	Glutathione Disulfide
LPO	Lipid peroxidation
NLRP3	NLR family pyrin domain containing 3
PyCARD	PYRIN domain and a caspase-recruitment domain
CASP1	gene that codes for Caspase-1
IL-1β	Interleukin 1 beta
IL-18	Interleukin-18
IFN-γ	Interferon gamma
TNF-α	Tumor necrosis factor alpha
Drp1	Dynamin-Related Protein 1
Acox1	Acyl-CoA oxidase 1
PMP70	Peroxisomal membrane protein 70
Pex5	Peroxisomal Biogenesis Factor 5
Pex19	Peroxisomal Biogenesis Factor 19
PPARα	Peroxisome proliferator-activated receptor alpha
CPT1α	Carnitine Palmitoyltransferase 1 alpha
ACSL1	Acyl-CoA Synthetase Long Chain Family Member 1
CoxIV	Cytochrome c oxidase subunit IV
SHP	Small Heterodimer Partner
FA	Fatty acid
TG	Triglyceride
ACC	Acetyl-CoA Carboxylase
FAS	Fatty Acid Synthase
SREBP-1c	Sterol Regulatory Element-Binding Protein-1c
CEBPA	CCAAT-Enhancer-Binding Protein Alpha
PPARG	Peroxisome Proliferator-Activated Receptor Gamma
SCD1	Stearoyl-CoA Desaturase 1
AGPAT1	1-acylglycerol-3-phosphate O-acyltransferase 1
DLK1	Delta-like 1 homolog
AMPK	AMP-activated protein kinase
p-mTOR	phosphorylated mTOR
